# Supplementary material for: Identification of Gene Responsible for Conferring Resistance against Race KN2 of Podosphaera xanthii in Melon
Source: Int J Mol Sci. 2024 Jan 17;25(2):1134. doi: 10.3390/ijms25021134 (PMC10816175; doi:10.3390/ijms25021134)
Supplement: Supplementary file 1 [file ijms-25-01134-s001.zip › ijms-2767539-supplementary.pdf]

Supplementary table S1: List of selected 23 LRR genes on chromosome 2.

No.	Gene ID	Location	Gene name	Product Size
1	MELO3C015287.2	562322..566608	F-box/LRR-repeat protein 15	997
2	MELO3C015517.2	2493916..2499670	F-box/LRR-repeat protein 15	527
3	MELO3C029319.2	4111584..4115605	NBS-LRR type resistance protein	511
4	MELO3C026254.2	26452836..26456497	Plant intracellular Ras-group-related LRR protein 1	727
5	MELO3C017212.2	25196362..25201792	Leucine-rich repeat (LRR) family protein	773
6	MELO3C029529.2	23497517..23506303	Plant intracellular Ras-group-related LRR protein 6	360
7	MELO3C017200.2	25285740..25289995	LRR receptor-like kinase family protein	662
8	MELO3C010220.2	15567693..15570245	LRR receptor-like kinase family protein	695
9	MELO3C026220.2	26629623..26632020	F-box/FBD/LRR-repeat protein At1g13570	479
10	MELO3C010203.2	15354458..15357541	LRR receptor-like kinase family protein	543
11	MELO3C029704.2	20465105..20466326	Plant intracellular Ras-group-related LRR protein 6	448
12	MELO3C015571.2	2968398..2970869	LRR receptor-like serine/threonine-protein kinase GSO1	591
13	MELO3C026278.2	26285991..26291000	Receptor protein kinase, putative	805
14	MELO3C015426.2	1568362..1569748	Leucine-rich repeat extensin-like protein 6	805
15	MELO3C025324.2	19339953..19342676	Leucine-rich repeat extensin-like protein 4	625
16	MELO3C015353.2	985162..987242	disease resistance protein RGA2-like	594
17	MELO3C010346.2	17481683..17485283	TMV resistance protein N	449
18	MELO3C010073.2	13110841..13113501	Leucine-rich receptor-like protein kinase family protein	759
19	MELO3C015573.2	2973017..2974918	receptor-like protein kinase BRI1-like 3	538

20	MELO3C015354.2	990582..993823	disease resistance protein RGA2-like	410
21	MELO3C015364.2	1092452..1093878	F-box protein family	683
22	MELO3C017502.2	22410210..22412711	PTI1-like tyrosine-protein kinase At3g15890	648
23	MELO3C010204.2	15363345..15367016	Receptor protein kinase CLAVATA1, putative	546

Supplementary table S2. Phenotyping data of F3 population against *P. xanthii* race KN2

**No.**	**Plants**	**Score**	**Phenotype**
P1	P1	1	R
P2	P2	5	S
F1	F1	1	R
1	SF2-1	2	S
2	SF2-2	2	S
3	SF2-3	4	S
4	SF2-4	4	S
5	SF2-5	2	S
6	SF2-6	5	S
7	SF2-7	1	R
8	SF2-8	2	S
9	SF2-9	1	R
10	SF2-10	2	S
11	SF2-11	5	S
12	SF2-12	1	R
13	SF2-13	3	S
14	SF2-14	5	S
15	SF2-15	5	S
16	SF2-16	1	R
17	SF2-17	1	R
18	SF2-18	3	S
19	SF2-19	5	S
20	SF2-20	5	S
21	SF2-21	1	R
22	SF2-22	1	R
23	SF2-23	3	S
24	SF2-24	1	R
25	SF2-25	5	S
26	SF2-26	1	R
27	SF2-27	1	R
28	SF2-28	1	R
29	SF2-29	1	R
30	SF2-30	2	S
31	SF2-31	1	R

32	SF2-32	4	S
33	SF2-33	4	S
34	SF2-34	2	S
35	SF2-35	3	S
36	SF2-36	1	R
37	SF2-37	1	R
38	SF2-38	1	R
39	SF2-39	5	S
40	SF2-40	2	S
41	SF2-41	3	S
42	SF2-42	4	S
43	SF2-43	1	R

Supplementary table S3. Markers and phenotype co-segregation against race KN2 in F3 population

F3 Plant	Phenotype	Score	MELO1	MELO6	MELO13
1	S	2	H	H	H
2	S	2	H	R	R
3	S	4	S	H	S
4	S	4	R	H	H
5	S	2	H	S	S
6	S	5	H	H	H
7	R	1	H	H	H
8	S	2	H	S	S
9	R	1	R	H	H
10	S	2	R	H	R
11	S	5	S	R	H
12	R	1	H	S	R
13	S	2	H	R	H
14	S	5	H	S	R
15	S	5	S	H	H
16	R	1	H	H	R
17	R	1	H	S	S
18	S	3	H	H	R
19	S	5	H	S	S
20	S	4	H	H	H
21	R	1	R	R	H
22	R	1	R	S	H
23	S	3	H	H	H
24	R	1	R	H	R
25	S	5	H	R	R
26	R	1	H	S	H
27	R	1	S	S	H
28	R	1	H	R	H
29	R	1	R	H	S
30	S	2	H	H	R

31	R	1	R	H	H
32	S	4	S	H	R
33	S	4	H	S	H
34	S	2	R	H	H
35	S	3	R	H	H
36	R	1	R	R	R
37	R	1	H	H	S
38	R	1	R	S	H
39	S	5	S	R	H
40	S	2	S	R	S
41	S	3	S	H	S
42	S	4	H	R	H
43	R	1	H	R	H
